# Type 1 Diabetes Mellitus Patients' Self-perception of Periodontal Diseases

**DOI:** 10.1055/s-0043-1772777

**Published:** 2023-12-04

**Authors:** Zaridah Zainal Abidin, Erni Noor, Noor Shafina Mohd Nor, Nor Shafina Mohamed Nazari, Azriyanti Anuar Zaini, Nurul Zeety Azizi, Shahrul Aiman Soelar, Marshah Mohamad Shahrizad, Rohaida Abdul Halim

**Affiliations:** 1 Centre of Paediatric Dentistry and Orthodontics Studies, Faculty of Dentistry, Universiti Teknologi MARA, Sungai Buloh, Selangor, Malaysia; 2 Centre of Studies for Periodontology, Faculty of Dentistry, Universiti Teknologi MARA, Sungai Buloh, Selangor, Malaysia; 3 Department of Paediatrics, Faculty of Medicine, Universiti Teknologi MARA, Sungai Buloh, Selangor, Malaysia; 4 Institute for Pathology, Laboratory and Forensic Medicine (I-PPerForM), Faculty of Medicine, Universiti Teknologi MARA, Sungai Buloh, Selangor, Malaysia; 5 Department of Restorative Dentistry, Faculty of Dentistry, Universiti Malaya, Kuala Lumpur, Malaysia; 6 Paediatric Department, Faculty of Medicine, Universiti Malaya, Kuala Lumpur, Malaysia; 7 Department of Paediatric Dentistry and Orthodontics, Faculty of Dentistry, Universiti Malaya, Kuala Lumpur, Malaysia; 8 Clinical Research Centre, Hospital Sultanah Bahiyah, Alor Setar, Kedah, Malaysia; 9 Kuching Division Dental Office, Sarawak State Dental Health Department, Braang Bayur Dental Clinic, Sarawak, Malaysia

**Keywords:** periodontal disease, type 1 diabetes mellitus, diabetes mellitus, oral health, self-perception

## Abstract

**Objectives** The study aimed to evaluate type 1 diabetes mellitus (T1DM) patients' self-perceived periodontal health status and to identify the association between periodontal disease (PD) and DM.

**Materials and Methods** This cross-sectional study included 113 T1DM children between 3 and 18 years old from the Universiti Teknologi MARA and the University of Malaya. Periodontal health parameters, including plaque index, gingival index, probing pocket depth, simplified basic periodontal examination, and clinical attachment loss, were recorded. Self-perceived periodontal health status was assessed with questionnaires.

**Statistical Analysis** Statistical analysis was performed to evaluate the sensitivity of the questionnaire and the relationship between T1DM and periodontal parameters.

**Results** The median age was 11.4 years. Half of them (50.4%) were females. A total of 83.5% rated their oral condition as good, whereas 27.5% reported a history of gingival bleeding. Clinical examination revealed that 48.7% had healthy gingiva, whereas 47.8% had gingivitis. The question “Do you have bleeding when brushing, flossing, or eating food?” showed good accuracy in the evaluation of PD ( *p* < 0.001).

**Conclusion** The questionnaire has a high potential to be used by medical professionals in identifying T1DM patients at risk of PD to guide nondental health care providers in making appropriate referrals to dental services.

## Introduction

Diabetes mellitus (DM) is a metabolic disease characterized by the dysregulation of carbohydrate metabolism. It commonly manifests as hyperglycemia due to diminished insulin secretion, impaired insulin action, or both. 1 Type 1 diabetes mellitus (T1DM) makes up 5 to 10% of all DM cases worldwide. 2 The disease stems from cell-mediated autoimmune destruction of the pancreatic β-cells that produce insulin. Depending on the degree of β-cell destruction, T1DM patients can experience reduced or absence of insulin secretion, as indicated by low or negligible levels of plasma C-peptide. The chronic hyperglycemic status can lead to long-term damage, dysfunction, and failure of different organs in DM patients, especially the eyes, kidneys, nerves, heart, and blood vessels. 3 Treatment modalities for T1DM include subcutaneous injections of insulin, maintenance of a healthy diet, and regular exercise. 4

Periodontal disease (PD) is a condition resulting from the infection and inflammation of the tooth-supporting tissues. 5 In the early stages of the disease, gingival inflammation may cause the gingival tissues to appear erythematous, or edematous, and potentially result in bleeding. Worse, gingivitis can progress into periodontitis that presents as attachment loss, alveolar bone resorption, and tooth mobility. 6 PD is one of the most common causes of tooth loss. 7 DM is associated with an increase in the prevalence, severity, and progression of periodontitis. 8 9 DM patients are three times more likely to suffer from periodontitis as compared with nondiabetics. 10 Furthermore, 10% of children with T1DM were reported to have higher rates of attachment loss and bone loss compared with their nondiabetic peers despite having comparable plaques score. 11 Moreover, a recent study showed that children with DM have twice the number of periodontal sites that eventually developed into periodontitis as compared with non-DM children. 12

The bidirectional association between DM and PD has been established in previous literature. 13 14 DM is known as one of the modifying factors of periodontitis that can accelerate the progress of PD 14 as DM can impair the periodontal tissue growth and matrix formation with fibroblasts, osteoblasts, and osteoclasts. 15 16 In addition, increased thickness of the gingival basement membrane in DM patients could also impair the vasculature of the periodontal tissues. 17 18 It has also been postulated that DM complicates PD by tipping the balance of oral microbiota, resulting in the dominance of periodontal pathogens. 19 20 However, more in-depth studies are needed to ascertain the differences in the biofilm of diabetics and nondiabetics. The latest evidence indicated that chronic inflammation in PD can aggravate complications of DM by worsening glycemic control.

The American Dental Association recommendation for diabetic patients is to receive medical follow-up on a 3-monthly basis. 21 During these visits, evaluation of hemoglobin A1c (HbA1c) and reassessment of diabetes management are recommended. However, T1DM patients with unsatisfactory glycemic control should be arranged for more frequent follow-ups to enhance their adherence to the treatment regime. During this quarterly visit, T1DM patients would be able to obtain the maximum benefits from the other multidisciplinary team of specialists, thus reinforcing good self-care practices, such as routine dental visit. 22 23 Apart from having known risk of PD, poor oral hygiene was observed in youngsters with T1DM especially those with uncontrolled HbA1c, which eventually could increase the risk of future oral disease(s). 24

In 2020, a study by Moore et al on professional health care workers in pediatric diabetes care teams showed that 76.2% of them were aware that periodontitis is a possible complication of diabetes. However, in 2022 Siddiqi and Zafar recorded a contradicting finding in which they found that the majority of medical practitioners (89%) were aware of the bidirectional association and knew that the glycemic index of patients with DM and suffering PDs could be improved by providing periodontal therapy. 25 However, as low as 4.8% had received training in recognizing patients who require dental care. This study reinforced the need for further training in this area to provide holistic care to DM patients. 26 27 There is an urgent need for accurate and reliable means of surveillance, detection, and diagnosis of periodontitis among children suffering from T1DM. In addition to that, referral to dental care professionals for appropriate and timely management is crucial, therefore appropriate screening tools for medical professionals to initiate referral of patients with DM is needed. 28 Hence, it is vital to establish an effective system for nondental health providers to identify individuals in need of dental care. 29 However, with limited resources available for regular screening and timely examination by dental practitioners, other options should be explored.

In 2022, a study by Mohd Said from Malaysia recommended the use of validated simplified digital periodontal health screening software for identification of PD at early stage in dental practice. 30 This identification tool appeared simple and appropriate to be used for screening of PDs. This showed an effort have been made to initiate the link from the general dental practice to specialist care. Nevertheless, the initiation from the medical practitioner has yet to be established. Hence, there is a need of questionnaires is of detecting populations at risk of oral diseases, which can be initiated from the medical professionals. On top of that, the accuracy and effectiveness of using a questionnaire when compared with the clinical examination ranged from moderate to high in various studies, thus indicating its potential to be can be administered to instil awareness, facilitate early detection, and predict the disease. 31 Therefore, in this context, a self- self-reported perception and the clinical parameters could contribute to the prevention and earlier diagnosis of PD, especially in individuals requiring complex clinical care. 32

Therefore, the use of adapted self-report measures for PD can be considered a low-cost alternative for the early detection and prevention of PD in DM patients. It can be beneficial for epidemiological studies and population surveillance of the periodontal condition. 33 This study aimed to compare the self-perceived periodontal health status using a guided questionnaire (GQ) with the clinical measurement by dental professionals among children and adolescents with T1DM. The study also set out to determine if the GQ is a valid tool to be used by nondental professionals to evaluate periodontal health status.

## Materials and Methods

This two-part study was a cross-sectional study conducted from October 2020 to May 2022 at two centers, Universiti Teknologi MARA (UiTM), Selangor, and the University of Malaya (UM), Kuala Lumpur. Content validity index for item (I-CVI) and face validity was conducted to validate the questionnaire. A reliability study of 20 participants and intraexaminer training and calibration was performed prior to the study.

All children and adolescents below 18 years old with T1DM diagnosis at UM and UiTM were invited to participate in the study. They must be able to communicate in English and or Bahasa Melayu. However, those undergoing active orthodontic therapy or using any antibiotics or medications in the last 3 months that might cause gingival alteration, such as drug-induced gingival enlargement, were excluded.

The sample size was determined using Epi-Info StatCal software based on the total number of the eligible T1DM patient ( *n* = 166) 34 and the prevalence of diabetes test knowledge was 50.4% (11.6/23 × 100). 35 Considering an attrition rate of 10% with a 95% confidence level and an acceptable margin of error of 5.6%, the final sample calculated was 118 (108 + 10%). A total of 113 T1DM patients were recruited. The participants and their parent(s) or caregiver(s) answered the questionnaire during their follow-ups. The questionnaire would be answered by parents of children below 16 years old. Subsequent appointments were arranged for the participants to undergo a dental examination.

## Definition

### Body Mass Index

Body mass index (BMI) was calculated as weight in kilograms divided by height in meters squared, i.e., BMI = weight (kg)/height2 (m ^2^ ). The calculated BMI was plotted on the Centers for Diseases and Prevention (CDC) BMI-to-age chart for the respective gender 36 ( Table 1 ).

**Table 1:** Body Mass Index classification

BMI classification	Definition
Underweight	<5th percentile
Normal weight	<85th centile
Overweigh	≥85th but <95th centile
Obesity	≥95th centile

Abbreviation: BMI, body mass index.

### Blood Pressure

In this study, blood pressure (BP) was reported as systolic (SBP) and diastolic (DBP) percentiles for age/sex/height. The classification is based on the individual's age plotted against the percentile of height in SBP or DBP (mm Hg) of the respective gender. 37 The height percentile was obtained using the CDC height-for-age percentile of the respective gender. Based on American Academy of Pediatrics, BP categories and stages are as follows 38 ( Table 2 ).

**Table 2:** Blood pressure classification

In children aged 1–13 y old	
BP classification	Definition
Normal	<90th percentile
Elevated	≥90th percentile to <95th percentile, or 120/80 mm Hg to <95th percentile (whichever is lower)
Stage 1 hypertension (HTN)	≥95th percentile to <95th percentile + 12 mm Hg, or 130/80–139/89 mm Hg (whichever is lower)
Stage 2 HTN	≥95th percentile + 12 mm Hg or ≥ 140/90 mm Hg (whichever is lower)
In children aged ≥ 13 y old	
BP classification	Definition
Normal	<120/< 80 mm Hg
Elevated	120/< 80–129/< 80 mm Hg
Stage 1 HTN	130/80–139/89 mm Hg
Stage 2 HTN	≥140/90 mm Hg

Abbreviations: BP, blood pressure; HTN, hypertension.

### Lipid Profile

According to the International Society for Pediatrics and Adolescent Diabetes and the American Diabetes Association recommended low-density lipoprotein cholesterol (LDL-C) of < 100 mg/dL (2.6 mmol/L) in youth with DM. 39

High-density lipoprotein cholesterol (HDL-C), total cholesterol, triglycerides can be categorized according to NHLBI 2011. 40

### Hemoglobin A1c

HbA1c is a glycoprotein formed by a direct reaction between blood glucose and hemoglobin. It is routinely in clinical research and clinical practice to evaluate diabetes control. For children, adolescents, and young adults ≤ 25 years old. With access to comprehensive care, HbA1c of < 53 mmol/dL (7.0%) is recommended. 41

## Study Instrument

### Guided Questionnaire

Self-reported questionnaires adopted from two recent studies were translated and validated to be used in this study. 42 43 The GQ consisted of 14 items with 8 items on patients' baseline characteristics (Part A) and 6 items on symptoms of PD (Part B). The GQ was used to screen for any PD comorbidity among the study participants.

### Clinical Examination

#### Simplified Basic Periodontal Examination

The simplified basic periodontal examination (sBPE) codes formed the basis of the assessment for patients under 18 years old. The examined teeth included one tooth from each sextant, i.e., the upper right six (tooth 16), the upper right one (tooth 11), upper left six (tooth 26), lower left six (tooth 36), lower left one (tooth 31), and lower right six (tooth 46). The WHO 621 probe with a light probing force of 20 to 25 g was used for this assessment. The sBPE codes were as follows: 0, healthy; 1, bleeding on gentle probing; 2, calculus present and/or plaque retention factors; 3, the presence of 4- to 5-mm pocket; and 4, the presence of 6 mm or more pocket; and *, furcation. 44

In children between 12 and 17 years with erupted permanent teeth, the full range of sBPE codes (0–4) was used. For children aged between 7 and 11 years with mixed dentition, the sBPE codes 0,1, and 2 were used. In the full primary dentition, sBPE similar to the one used for children as young as 3 years of age was performed. 45 Apart from that, plaque index (PI) 46 and gingival index (GI) 47 were also assessed.

### Statistical Analysis

Data analysis was performed using the Statistical Package for the Social Sciences Version 20.0. Association between the GQ and the clinical examination was assessed using the Pearson chi-square test and Fisher's exact test. A *p* -value < 0.05 was considered statistically significant. Diagnostic tests (sensitivity, specificity, accuracy, positive predictive value, negative predictive value, and receiving operating characteristic curve) were performed to measure the performance of each question with the basic periodontal examination (BPE) as the reference for the periodontal evaluation. For this purpose, the periodontal status was dichotomized as “0” for healthy and “1 and above” for having PD.

## Results

Questionnaire I-CVI value 0.96 indicate high value of all items. And, following changes made based on expert opinion, a good face validation could be seen. Intraexaminer training and calibration (intraclass correlation coefficient revealed a good value of 85%) with acceptable reliability Cronbach's α of 0.77. Table 3 outlines the sociodemographic, clinical parameters, and oral hygiene care characteristics of the participants. From the 113 participants, 24.8% ( *n* = 28) presented with stage 1 hypertension, whereas another 18.6% ( *n* = 21) had elevated BP. Despite only 6.2% of the participants being obese, as high as 92% of them recorded uncontrolled HbA1c. Similarly, 19.5% of the participants were found to have high LDL-C despite normal body weight.

**Table 3:** Sociodemographic, clinical parameters, and oral hygiene care of participants

Variables	*n*	(%)
Sex		
Male	56	(49.6)
Female	57	(50.4)
Age		
< 6 y	16	(14.2)
7–12 y	39	(34.5)
13–18 y	58	(51.3)
Race		
Malay	55	(48.7)
Chinese	27	(23.9)
Indian	30	(26.5)
Others	1	(0.9)
Blood pressure 38		
Normal	64	(56.6)
Elevated	21	(18.6)
Stage 1 hypertension	28	(24.8)
FBG 41		
Normal fasting glucose	54	(47.8)
Impaired fasting glucose	12	(10.6)
Diabetes fasting glucose	47	(41.6)
RBG 41		
Normal glucose tolerance	11	(9.7)
Impaired glucose tolerance	39	(34.5)
Diabetes glucose tolerance	63	(55.8)
BMI 36		
Underweight	11	(9.7)
Healthy weight	68	(60.2)
Overweight	27	(23.9)
Obesity	7	(6.2)
HbA1c 41		
Controlled	9	(8.0)
Uncontrolled	104	(92.0)
Lipid profile		
TG 40		
Acceptable	30	(26.5)
Borderline	24	(21.2)
High	41	(36.3)
Missing	18	(15.9)
TC 40		
Acceptable	31	(27.4)
Borderline	34	(30.1)
High	30	(26.5)
Missing	18	(15.9)
LDL-C 39		
Acceptable	48	(42.5)
Borderline	25	(22.1)
High	22	(19.5)
Missing	18	(15.9)
HDL-C 40		
Low	13	(11.5)
Borderline	6	(5.3)
Acceptable	75	(66.4)
Missing	19	(16.8)
Duration of diabetes		
< 5 y	66	(58.4)
5–10 y	38	(33.6)
> 10 y	9	(8.0)
Frequency of brushing		
Morning	17	(15.0)
Morning/before going to sleep	72	(63.7)
Morning/before going to sleep and after eating food	22	(19.5)
Use flossing	1	(0.9)
Others/adjunct	1	(0.9)

Abbreviations: BMI, body mass index; FBG, fasting blood glucose; HbA1c, hemoglobin A1c; HDL-C, high-density lipoprotein cholesterol; LDL-C, low-density lipoprotein cholesterol; RBG, random blood glucose; TC, total cholesterol; TG, triglycerides.

Further analysis revealed that 40.9 and 53.8% of participants with healthy BMI exhibited high LDL-C and low HDL-C, respectively ( Table 4 ). In addition, uncontrolled HbA1c was observed among 91.2% of participants despite normal BMI. A higher percentage of uncontrolled HbA1c was also reported among participants with acceptable LDL-C (87.5%), acceptable HDL-C (90.7%), and less than 5 years of DM (90.9%). However, all these associations were not statistically significant ( *p* > 0.05) ( Table 5 ).

**Table 4:** Association between low-density lipoprotein cholesterol, high-density lipoprotein cholesterol, and body mass index

Variables	Underweight		Healthy weight		Overweight		Obesity		*p* -Value
	*n*	(%)	*n*	(%)	*n*	(%)	*n*	(%)	
LDL-C									
Acceptable	2	(4.2)	33	(68.8)	10	(20.8)	3	(6.3)	0.180 a
Borderline	2	(8.0)	17	(68.0)	6	(24.0)	0	(0.0)	
High	1	(4.5)	9	(40.9)	10	(45.5)	2	(9.1)	
HDL-C									
Low	0	(0.0)	7	(53.8)	4	(30.8)	2	(15.4)	0.392 a
Borderline	1	(16.7)	3	(50.0)	2	(33.3)	0	(0.0)	
Acceptable	4	(5.3)	48	(64.0)	20	(26.7)	3	(4.0)	

Abbreviations: BMI, body mass index; HDL-C, high-density lipoprotein cholesterol; LDL-C, low-density lipoprotein cholesterol.

a Fisher's exact test.

**Table 5:** Association between selected variables and hemoglobin A1c

Variables	Controlled		Uncontrolled		*p* -Value
	*n*	(%)	*n*	(%)	
BMI					
Underweight	1	(9.1)	10	(90.9)	1.000 a
Healthy weight	6	(8.8)	62	(91.2)	
Overweight	2	(7.4)	25	(92.6)	
Obesity	0	(0.0)	7	(100.0)	
LDL-C					
Acceptable	6	(12.5)	42	(87.5)	0.270 a
Borderline	2	(8.0)	23	(92.0)	
High	0	(0.0)	22	(100.0)	
HDL-C					
Low	1	(7.7)	12	(92.3)	1.000 a
Borderline	0	(0.0)	6	(100.0)	
Acceptable	7	(9.3)	68	(90.7)	
Duration of T1DM					
< 5 y	6	(9.1)	60	(90.9)	0.647 a
5–10 y	2	(5.3)	36	(94.7)	
> 10 y	1	(11.1)	8	(88.9)	

Abbreviations: BMI, body mass index; HDL-C, high-density lipoprotein cholesterol; LDL-C, low-density lipoprotein cholesterol; T1DM, type 1 diabetes mellitus.

a Fisher's exact test.

Table 6 shows that 27.5% of participants who reported bleeding from gingiva upon brushing, flossing, and eating were found to have BPE during the clinical examination. This finding indicated a high sensitivity (50%) and specificity of the questionnaire (94.5%). In addition, 90% of T1DM presented with gingival bleeding were at risk of having PD ( *p* < 0.001).

**Table 6:** Frequency of responses for periodontal health questionnaire and diagnostic tests for each question in relation to simplified basic periodontal examination

Questions	Answer	*n* (%)	SS	SP	ACC	PPV	NPV	AUC	*p* -Value
Part A 1.Do you think you have gum disease?	Yes No	14 (12.8) 95 (87.2)	16.7	90.9	54.1	64.3	52.6	0.538	0.495
2.Overall, how do you rate your teeth and gum health?	Good Bad	91 (83.5) 18 (16.5)	20.4	87.3	54.1	61.1	52.7	0.538	0.492
3.Have you ever had gum treatment for gum disease, such as scaling either above or below gum?	Yes No	19 (17.4) 90 (82.6)	22.2	87.3	55.0	63.2	53.3	0.547	0.393
4.Have you ever had loose teeth without injury?	Yes No	2 (1.8) 107 (98.2)	1.9	98.2	50.5	50.0	50.5	0.500	0.998
5.Have you ever been told by a dental professional that you have gum disease?	Yes No	1 (0.9) 108 (99.1)	0.0	98.2	49.5	0.00	50.0	0.491	0.870
6.During the past 3 mo, have you ever noticed that your gum doesn't look good?	Yes No	7 (6.4) 102 (93.6)	11.1	98.2	55.0	85.7	52.9	0.546	0.403
7.Did you use dental floss or “other devices” for tooth cleaning in the last 7 d?	Yes No	9 (8.3) 100 (91.7)	11.1	94.5	53.2	66.7	52.0	0.528	0.611
8.Did you use mouthwash or other dental rinses for “dental problems” treatment in the last 7 d?	Yes No	13 (11.9) 96 (88.1)	14.8	90.9	53.2	61.5	52.1	0.529	0.606
Part B Do you have the following symptoms?									
1. Bleeding when brushing, flossing, or eating food	Yes No	30 (27.5) 79 (72.5)	50.0	94.5	72.5	90.0	65.8	0.723	<0.001
2. Swelling, red, or painful gums for no apparent reason	Yes No	9 (8.3) 100 (91.7)	13.0	96.4	55.0	77.8	53.0	0.547	0.401
3. Teeth look longer, and the smile appears more “toothy”	Yes No	3 (2.8) 106 (97.2)	0.0	94.5	47.7	0.0	49.1	0.473	0.623
4. Bad breath/ halitosis/foul mouth odor	Yes No	11 (10.1) 98 (89.9)	14.8	94.5	55.0	72.7	53.1	0.547	0.400
5. Loosening or shifting of teeth in the affected area	Yes No	4 (3.7) 105 (96.3)	3.7	96.4	50.5	50.0	50.5	0.500	0.995
6. Pus oozing between the teeth	Yes No	0 (0.0) 109 (100.0)	0.0	100.0	50.5	0.0	50.5	0.500	1.000

Abbreviations: ACC, accuracy in percentage; AUC, area under the curve; NPV, negative predictive value in percentage; PPV, positive predictive value in percentage; SP, specificity in percentage; SS, sensitivity in percentage.

As presented in Table 7 , the mean PI and GI were 0.37 ± 0.31 and 0.27 ± 0.32, respectively. In other words, 48.7% of the participants had healthy periodontal status. Among 47.8% of participants with unhealthy gingiva, 7% of them were found to have periodontitis.

**Table 7:** Clinical parameters of the periodontal health status of participants

Variables	*n*	(%)	Mean	Standard deviation
Index				
Plaque index	109	96.5	0.37	0.31
Gingival index	109	96.5	0.27	0.32
Missing	4	3.5		
Periodontal status				
Healthy	55	48.7		
Gingivitis	54	47.8		
Missing	4	3.5		
Periodontitis				
Nonperiodontitis	105	92.9		
Periodontitis	4	3.5		
Missing	4	3.5		

## Discussion

PD is the sixth most common complication among DM patients. 48 As T1DM patients face an increased risk of PD, 49 it is vital to improve the awareness of medical professionals and patients on the prevention and identification of oral diseases. Medical–dental coordinated care needs to be strengthened for this purpose. The use of self-reported questionnaires can increase T1DM patients' awareness and self-perception of oral diseases. During most medical consultations, physicians and other nonoral health professionals often overlook the need for oral clinical examination. 32 Hence, the use of a self-reported questionnaire can also evoke their attention to DM patients at risk of PD. Following that, health care providers can refer the patients to a dentist for further management to prevent oral diseases. 50

In this study, it was found that respondents who answered “yes” to the question “Do you have bleeding when brushing, flossing, or eating food?” were associated with a high score of BPE compared with those who answered “no.” The sum of sensitivity plus specificity for this item was 144%, which is considered as a “good validity.” 33 Agreed by study in Japan population, inclusion of the question on gum bleeding would improve the predictive performance of the questionnaire as it is less confusing than other items. 51 This finding also echoed a few other studies in which the term gum bleeding should be used rather than gingivitis when interacting with patients 52 53 54 as not all may be familiar with the dental term of gingivitis, especially children and adolescents. In addition, Elhassan et al (2017) also suggested that patients could relate to bleeding better than the appearance of swollen gum. 55

According to the Nelson's validity classification, self-reported PD (painful gums, tooth mobility, and people's opinions whether they have gums disease) can be classified as having moderate to high validity. 33 56 However, in our study, the items “Do you think you have gum disease,” “Swelling, red, or painful gums for no apparent reason,” and “Loosening or shifting of teeth in the affected area” were found to have low sensitivity of 16.7, 13, and 3%, respectively. The reason probably due to this set of questionnaire has good predictive ability for periodontitis, especially in the severe cases rather than gingivitis alone, as only 3.5% patients in our study presented with periodontitis. 51 Supported by one study where the prevalence of periodontitis is high, the predictive performance of similar self-reported questions presented reported to be more accurate. 57

In this study, the questionnaire on the perception of oral health for participants aged less than 16 years old (72.6%) was answered by their parents or caregiver. With a low sensitivity (18.7%) and high specificity (86%), it can be regarded as having parents may have good perception of the child's oral health rather than poor perception. This finding agrees with previous studies in which parental perception of their children's oral status was found to be superior to the clinical findings. 58 59 60 61 In addition, parental perceptions of oral health are often dependent on clinical conditions with recognizable symptoms, such as dental caries with toothache rather than other less obvious oral conditions such as gum problems, malocclusion, or dental trauma. 62 Similarly, in an earlier study by Cyrino et al (2011), the patient's perception of health was found to be better than the actual presentation of the disease. 63 The remaining 27.4% of the questionnaire answered by 16- and17-year-olds showed slightly higher sensitivity (27.3%) and specificity (100%). More studies reported that younger individuals may not be able to identify PD. 32 64 However, contradicting to the statement, adult age more than 60 were less likely to report gingival bleeding symptom correctly compared with less than 40 years old, which probably due to more serious manifestation occur rather than gingival bleeding. 65

According to a new classification of PD, 3.5% or four patients in this study had periodontitis. 66 All four participants answered “yes” for gum bleeding even though only three of them claimed that they have gum disease. One of them with an advanced stage (stage III) of gum disease answered “yes” to people's opinions on whether they have gum disease. The individual also answered “yes” for bad gum condition, painful swollen gums, and tooth mobility. This means that, our findings agree that more value was seen pertaining to self-reported PD if severe stage periodontitis was encountered. 67 Thus, this questionnaire would be able to exclude healthy individuals from periodontal clinical examination at a cheaper cost and would be an alternative of gold standard periodontal examination in cost-limited epidemiology survey. 68 More importantly, this can expedite the early diagnosis and treatment of PD.

The HbA1c of most participants showed uncontrolled T1DM as many of them did not achieve the target HbA1c of < 53 mmol/mol (<7.0%) for children and adolescents who have access to comprehensive care. 41 In this study, almost half of the patients were diagnosed with gingivitis (47.8%). However, there was no significant association between HbA1c and gingivitis ( *p* = 0.271). In contrast, a significant correlation was detected between PI and sBPE ( *p* < 0.00). This shows that periodontal condition was more associated with the presence of visible plaque rather than the underlying metabolic status. According to a recent study, HbA1c in children and adolescent has a low correlation with the gingival condition compared with adult DM patients. 69

Even though the prevalence of T1DM in children and adolescents is increasing, 70 we faced certain challenges in recruiting eligible patients during the pandemic in view of limited follow-up appointments available during the lockdown period. Furthermore, more than 10 patients or their parents declined to participate.

## Conclusion

This study concluded that the GQ has a high potential to be used in identifying T1DM children at a greater risk for PD and in need of an oral examination. It can be adopted as a tool for nondental health care providers to screen for PD before making appropriate dental referrals for the patients. In the long term this will ensure a seamless and coordinated care pathway for DM patients requiring dental care
